# Non-Transfusional Hemocomponents: From Biology to the Clinic—A Literature Review

**DOI:** 10.3390/bioengineering5020027

**Published:** 2018-03-31

**Authors:** Roberta Gasparro, Erda Qorri, Alessandra Valletta, Michele Masucci, Pasquale Sammartino, Alessandra Amato, Gaetano Marenzi

**Affiliations:** 1 Department of Neuroscience, Reproductive Science and Dental Science, University of Naples Federico II, 80131 Naples, Italy; alessandra.valletta@unina.it (A.V.); michelemasucci@hotmail.com (M.M.); aale.amato@gmail.com (A.A.); gaetano.marenzi@gmail.com (G.M.); 2 Division of Oral and Maxillofacial Surgery, Department of Dentistry, Albanian University, 1000 Tirana, Albania; e.qorri@albanianuniversity.edu.al; 3 Multidisciplinary Department of medical-Surgical and Dental Specialities, University of Naples “Luigi Vanvitelli”, 80136 Italy; pasqualesammartino91@gmail.com

**Keywords:** platelet, growth factors, PRP, tissue regeneration

## Abstract

Non-transfusional hemocomponents for surgical use are autogenous products prepared through the centrifugation of a blood sample from a patient. Their potential beneficial outcomes include hard and soft tissue regeneration, local hemostasis, and the acceleration of wound healing. Therefore, they are suitable for application in different medical fields as therapeutic options and in surgical practices that require tissue regeneration.

## 1. Introduction

Non-transfusional hemocomponents for surgical use are innovative tools of regenerative medicine and are widely used in clinical and surgical practices that require tissue regeneration [1,2]. Their potential beneficial outcomes include hard and soft tissue regeneration [3], local hemostasis [4] and the acceleration of wound healing [5]. Therefore, they are suitable for application in different medical fields as therapeutic options [6,7,8].

## 2. Definition and History

Non-transfusional hemocomponents for surgical use are autogenous products prepared through the centrifugation of a blood sample from a patient [9]. These preparations can be solutions or gels and can be injected or placed in a surgical site in order to regenerate the damaged tissues [1]. In transfusion medicine, a specific platelet concentrate was originally named “platelet-rich plasma” (PRP), as it describes a blood component that contains high levels of platelets prepared by using a centrifuge to separate the platelet-rich fraction from the whole blood. It found application in the treatment and prevention of hemorrhages in thrombocytopenic patients [10]. PRP is defined as the volume of the plasma fraction from autologous blood having a platelet concentration above the baseline (200,000 platelets/µL) [11]. In particular, for example, the advantageous biological effects on bone regeneration seem to occur when PRP with a platelet concentration of approximately 1,000,000/µL is used [12].

The first topical application of platelet gel, called a “platelet–fibrinogen–thrombin mixture,” was that used by Rosenthal for corneal adhesion [13] and for the sealing of perforating corneal wounds in rabbits [14]. Subsequently, it was used, with the name “platelet–fibrinogen–thrombin adhesive,” to reduce the haemorrhage related to microvascular anastomosis in a rat model [15] and to repair cerebrospinal fistulas in dogs [16].

Several years later, these products were considered not only as fibrin tissue adhesives but also as applications having direct healing properties. In fact, Knighton and collaborators [17] used platelet concentrates containing, for example, “platelet derived wound healing factors” (PDWHF) for the treatment of chronic, non-healing cutaneous ulcers. 

Finally, in 1998, Marx reintroduced the concept and the term PRP to define an autologous source of platelet-derived growth factor and transforming growth factor beta and used it in maxillofacial bone reconstruction [3].

A few years afterwards, a second generation of platelet concentrates was introduced by Choukroun, named “platelet-rich fibrin” (PRF), and this started to replace the use of PRP in oral and maxillofacial surgery [18].

## 3. Classification and Techniques

Several techniques for platelet concentrates are available, so there are various different products with different biological features and clinical uses [19].

The methods available can be classified into four main categories: pure PRP (P-PRP), leucocyte-rich PRP (L-PRP), pure PRF (P-PRF), and leucocyte-rich PRF (L-PRF) [20].

In brief, in the PRP techniques, the blood is collected with anticoagulants and immediately processed by centrifugation. A first centrifugation separates the blood into three layers, red blood cells (RBCs) at the bottom, and acellular plasma (PPP, platelet-poor plasma) at the top, with a “buffy coat” layer appearing in between, in which the platelets are concentrated. A second step discards both the RBC and PPP layers to collect only the “buffy coat.” Finally, the obtained platelet concentrate is activated with thrombin and/or calcium chloride (or similar factors) to trigger the platelet activation and fibrin polymerization.

Although the methods for platelet gel preparation are similar, there are different practices relating to the growth factor recovery and the kinetics of its release from the gel [11]. 

In Choukroun’s PRF, the blood is collected without any anticoagulant and immediately centrifuged in a single step that allows for the formation of an L-PRF clot. This product does not need any activation since no thrombin or calcium chloride is required, making the procedure simpler and easier for clinicians to use. 

**Figure 1:**
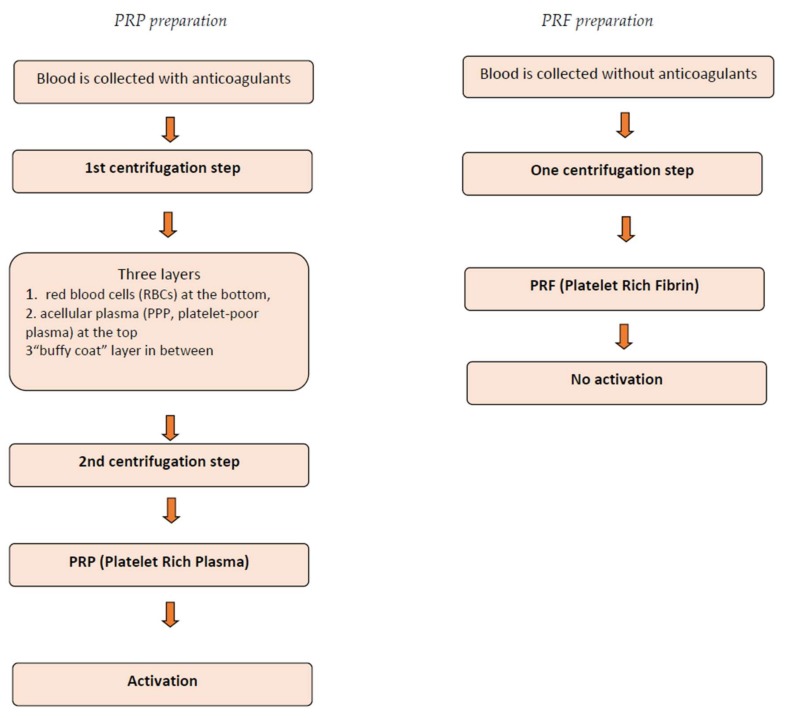
Main phases of platelet rich plasma (PRP) and platelet rich fibrin (PRF) preparation.

Figure 1 summarizes the main phases of PRP and PRF preparation.

## 4. Biological Behaviour

A wide number of events and various signalling proteins mediate and regulate the healing process of tissues.

During tissue repair and regeneration in vivo, the platelets release high concentrations of proteins, such as growth factors (GFs) and other substances [21].

Platelet granules represent a source of cytokines, like RANTES or CCL5, and GFs involved in cell proliferation and differentiation, wound healing, and tissue repair [22,23,24].

Leucocytes are also a significant source of cytokines and GFs, which interact with those released by the platelets [25].

The main GFs involved in tissue repair and their functions are as follows: platelet-derived GF (PDGF), vascular endothelial GF (VEGF), epidermal GF (EGF), transforming GF β (TGF-β), insulin like GF-1 (IGF-1), and fibroblast GF (FGF).

It has been proven that L-PRF membranes slowly release significant amounts of certain GFs (TGFβ-1, PDGF-AB, and VEGF) and thrombospondin-1 (TSP-1), a matrix protein, over a period of at least seven days [26].

Different releases of these factors have been found in the literature.

Comparing PRF and PRP, several inflammatory cytokines, such as IL-6, IL-8, IL-10, IFN-γ, MIP-1a, MIP-1b, and TNFα, have been found in higher concentrations in PRF. In contrast, the levels of RANTES, a chemokine released mainly by the platelets, were three-fold higher in PRP. Instead, the concentration of GFs VEGF and TGFb1 was higher, respectively, in PRF compared to PRP. Finally, the amount of PDGF was two-fold lower in PRF compared to PRP [27].

If a comparison is made in relation to the release of cytokines/chemokines and GFs from clots from original L-PRF and modified A-PRF (advanced-PRF), an experimental low-force modified procedure, it can be noted that A-PRF collected the same content of leukocytes and released a similar amount of inflammatory cytokines, but secreted higher levels of Eotaxin, CCL5, PDGF, and VEGF [28], rendering this a promising product for the future. 

PRP storage, like freezing/thawing, is also a hot topic in the literature. Although in some studies, no differences were found in the releasing of VEGF [29], frozen PRP showed a lower GF release from platelets with respect to that of the fresh preparation, but preserved the biological activities in chondrocyte and synoviocyte cultures [30]. On the contrary, freeze-dried PRP was found more effective in the acceleration of bone healing compared to that of fresh PRP in a rat model [31]. 

## 5. Clinical Applications of Platelet Concentrates

### 5.1. Reconstructive and Implant Surgery

In bone graft, implant and reconstructive surgery, the analysis of the literature does not provide a definitive answer [32]. The first application of the topical use of platelet concentrates in oral surgery occurred about 20 years ago [3,4,5,6,7,8,9,10,11,12,13,14,15,16,17,18,19,20,21,22,23,24,25,26,27,28,29,30,31,32,33]. PRP was added to bone grafts in reconstructive surgery and a higher radiographic maturation rate than that of the grafts without PRP was found. In addition, a greater bone density in the grafts with the PRP than in the grafts without any PRP was demonstrated [3].

However, on the contrary, some authors have concluded that PRP gels have no impact on bone regeneration, either alone or in association with bone grafts [34,35]. 

In implant surgery, PRF membranes have been used to cover the head of the implants and thus act as a fibrin bandage between the allograft and the gingival tissue [36]. Moreover, the use of PRF led to a substantial thickening of the keratinized gingival tissue around the implants, playing a significant role in enhancing the stability of the grafted bone surface [37] and in determining the final result of prosthodontic rehabilitation, improving the aesthetic integration [38]. 

PRF seems to reduce post-operative pain and edemas and to limit infections. Thus, the control of inflammation seems to be another advantage resulting from the use of PRF during bone grafting [37].

### 5.2. Prevention of Hemorrhagic Complications after Dental Extraction

Platelet hemocomponents have been used to prevent post-operative hemorrhagic complications in dental extractions in heart surgery patients treated with artificial mechanical heart valves [39]. For example, PRP gel placed in the alveolar socket after extraction without any heparin administration after the suspension of oral anticoagulant drugs allowed for an adequate hemostasis after the dental extraction [40]. 

In the same way, the application of L-PRF clots significantly reduced bleeding after dental extractions without any suspension of the continuous oral anticoagulant therapy in heart surgery patients [4].

Therefore, these platelet hemocomponents can be safely used as local hemostatic agents, replacing fibrin glue and similar products and reducing the costs of the procedure.

### 5.3. Periodontology

The effect of platelet concentrates in periodontology is controversial.

PRP has been used to induce and accelerate bone regeneration in the treatment of periodontal defects at the distal root of the mandibular second molar after the surgical extraction of a mesioangular impacted mandibular third molar. A notable reduction in the probing depth and an improvement in the probing attachment level have been found [41].

Comparing intra-bony defects treated either with autologous PRF or a conventional open flap debridement alone, there was a greater reduction in probing depth (PD), greater clinical attachment level (CAL) gain, and greater intra-bony defect filling at sites treated with PRF [42].

In the treatment of gingival recession, Jancovic and collaborators showed that the use of PRF membranes provided acceptable clinical results, promoting enhanced wound healing and decreased patient discomfort compared to connective tissue grafts (CTGs). However, a greater gain in keratinized tissue width was obtained in the CTGs compared to the PRF group [43].

### 5.4. Orthopedic and Sports Medicine

There is no unanimous agreement in sports medicine on the selection of the most appropriate technique for the preparation of specific platelet concentrates, particularly in relation to the exact cell content of the injectable platelet product [7,8,9,10,11,12,13,14,15,16,17,18,19,20,21,22,23,24,25,26,27,28,29,30,31,32,33,34,35,36,37,38,39,40,41,42,43,44].

Preclinical studies support the use of PRP for the treatment of tendon injuries and disorders [45], ligament injuries, and muscle injuries [46]. 

Moreover, PRP can stimulate chondral anabolism and reduce catabolic processes, and may improve joint homeostasis reducing synovial membrane hyperplasia in osteoarthritis [47].

### 5.5. Plastic Surgery and Dermatology

PRP has been successfully used to improve wound healing. The underlying biological mechanisms are related to the improved proliferation of endothelial cells and vascularization and the stimulating effects on the formation of granulation tissue [48].

Platelet gels have improved not only the practice of wound healing when used for skin chronic ulcers but also the time required for healing and hospitalization, which has led to a decrease in morbidity and health costs [49,50].

More recently, interest has been increasing in the application of PRP in dermatology, particularly in skin rejuvenating effects [48]. It has also been trialed as a new therapy for androgenetic alopecia (AGA), showing increased hair density compared with controls [51], and a clinical improvement in the mean number of hairs and in the number of hair follicles two weeks after PRP treatment [52].

Table 1 summarizes the main articles included in this literature review.

## 6. Conclusions

Non-transfusional hemocompoents are innovative tools in regenerative medicine and are widely used for clinical and surgical practices that require tissue repair or regeneration. Studies of blood-derived biomaterials are increasing in efforts to create different therapeutic formulations that can adapt to the needs of various biomedical applications, including orthopedic and maxillofacial surgery, sports medicine, bone reconstruction, tissue engineering, and cosmetic and dental implant surgery. The authors’ efforts will be directed toward research and clinical trials under rigid protocols to improve the effects of these products, especially in regenerative procedures.

## Figures and Tables

**Table 1:** Main articles included in the literature review.

References	Study Type	Results
**Hard and soft tissue regeneration**		
Marx R.E. et al., 1998 [3]	Clinical study	PRP enhanced bone graft
Weibrich G. et al., 2004 [12]	Clinical study	PRP was not beneficial in accelerating osseointegration
Broggini N. et al., 2011 [34]	Histological study	PRP did not lead to greater bone remodeling
Torres J. et al., 2008 [35]	Morphometric study	PRP was not beneficial in osseous regeneration
Simonpieri et al, 2009 [36]	Clinical study	PRF was helpful for periosteum healing and maturation
Sammartino et al., 2005 [41]	Clinical study	PRP was effective in accelerating bone regeneration
Thorat M. et al., 2011 [42]	Clinical study	PRF improved intra-bony defect fill
Sommeling et al., 2013 [48]	Systematic review	PRP enhanced bone graft regeneration
**Hemostasis**		
Della Valle A. et al., 2003 [40]	Clinical study	PRP reduced postoperative hemorrhage
Sammartino G. et al., 2011 [4]	Clinical study	PRF reduced postoperative hemorrhage
**Wound healing**		
Picard F. et al., 2015 [5]	Literature Review	PRP may be beneficial in diabetic chronic wounds
Knighton D.R. et al., 1986 [17]	Clinical study	PDWHF promoted the healing of chronic cutaneous ulcers
Jankovic S. et al., 2012 [43]	Clinical study	PRF enhanced wound healing in gingival recession
Sommeling et al., 2013 [48]	Systematic review	PRP improved the wound healing rate
Mazzucco et al., 2004 [49]	Clinical study	Platelet gel improved chronic unhealing wounds
Saad Setta et al., 2011 [50]	Clinical study	PRP enhanced healing of chronic diabetic foot ulcers

PRP: platelet rich plasma; PRF: platelet rich fibrin; PDWHF: platelet-derived wound healing factors.
